# Validation of a new prognostic model to predict short and medium-term survival in patients with liver cirrhosis

**DOI:** 10.1186/s12876-020-01407-8

**Published:** 2020-08-12

**Authors:** Tomasz Dziodzio, Robert Öllinger, Wenzel Schöning, Antonia Rothkäppel, Radoslav Nikolov, Andrzej Juraszek, Paul V. Ritschl, Martin Stockmann, Johann Pratschke, Maximilian Jara

**Affiliations:** 1 Charité – Universitätsmedizin Berlin, corporate member of Freie Universität Berlin, Humboldt-Universität zu Berlin, and Berlin Institute of Health, Department of Surgery - Campus Charité Mitte / Campus Virchow-Klinikum, Augustenburger Platz 1 |, 13353 Berlin, Germany; 2 Evangelisches Krankenhaus Paul Gerhardt Stift, Department of General, Visceral and Vascular Surgery, Lutherstadt Wittenberg, Germany

**Keywords:** End-stage liver disease, LiMAx®, MELD, Mortality risk, Prognosis, Survival, Survival scores

## Abstract

**Background:**

MELD score and MELD score derivates are used to objectify and grade the risk of liver-related death in patients with liver cirrhosis. We recently proposed a new predictive model that combines serum creatinine levels and maximum liver function capacity (LiMAx®), namely the CreLiMAx risk score. In this validation study we have aimed to reproduce its diagnostic accuracy in patients with end-stage liver disease.

**Methods:**

Liver function of 113 patients with liver cirrhosis was prospectively investigated. Primary end-point of the study was liver-related death within 12 months of follow-up.

**Results:**

Alcoholic liver disease was the main cause of liver disease (*n* = 51; 45%). Within 12 months of follow-up 11 patients (9.7%) underwent liver transplantation and 17 (15.1%) died (13 deaths were related to liver disease, two not). Measures of diagnostic accuracy were comparable for MELD, MELD-Na and the CreLiMAx risk score as to power in predicting short and medium-term mortality risk in the overall cohort: AUROCS for liver related risk of death were for MELD [6 months 0.89 (95% CI 0.80–0.98) *p* < 0.001; 12 months 0.89 (95% CI 0.81–0.96) *p* < 0.001]; MELD-Na [6 months 0.93 (95% CI 0.85–1.00) *p* < 0.001 and 12 months 0.89 (95% CI 0.80–0.98) *p* < 0.001]; CPS 6 months 0.91 (95% CI 0.85–0.97) *p* < 0.01 and 12 months 0.88 (95% CI 0.80–0.96) *p* < 0.001] and CreLiMAx score [6 months 0.80 (95% CI 0.67–0.96) *p* < 0.01 and 12 months 0.79 (95% CI 0.64–0.94) *p* = 0.001]. In a subgroup analysis of patients with Child-Pugh Class B cirrhosis, the CreLiMAx risk score remained the only parameter significantly differing in non-survivors and survivors. Furthermore, in these patients the proposed score had a good predictive performance.

**Conclusion:**

The CreLiMAx risk score appears to be a competitive and valid tool for estimating not only short- but also medium-term survival of patients with end-stage liver disease. Particularly in patients with Child-Pugh Class B cirrhosis the new score showed a good ability to identify patients not at risk of death.

## Background

Mathematical scores are frequently used tools of clinical routine frequently used in the assessment of disease severity and in the determination of patient prognosis [1–4]. Historically the Child-Pugh score (CPS) was implemented as a clinical score to evaluate prognosis in liver cirrhosis [1]. Initially CPS was used as the basis for prioritizazion of organs for liver transplantation. Since 2002, the model for end-stage liver disease (MELD) has become the reference mathematical model for many liver transplant societies to facilitate and objectivize the allocation of donor organs to liver transplant candidates. Nevertheless, there is an ongoing discussion on the prognostic value of both scores [5]. In particular, the known disparities of MELD based organ allocation systems favorizing a “sickest first” policy, suggest that room remains for improvement [6].

In patients with liver cirrhosis the predictive power of quantitative liver function tests has been reported with results comparable to established biological indices in several studies [7, 8]. Here, ^13^C-liver function breath tests were identified as particularly accurate [9, 10]. In our past studies we extensively evaluated the ^13^C-methacetin LiMAx® methodology and its prognostic value in different clinical situations [11–14].

The results of our prior studies indicated that enzymatic liver function and serum creatinine are independent factors for short-term survival of patients with liver cirrhosis. In a cohort study we prospectively investigated the ability of the LiMAx®-test to predict 3-month mortality in patients with liver cirrhosis [15]. Here, the LiMAx® score and serum creatinine were independent predictors for liver-failure related death in a cox proportional-hazards model. Furthermore, the logistic regression analysis identified the LiMAx® score and serum creatinine as independent factors of mortality. On the basis of these results we have proposed a new score, combining the predictive power of both, the LiMAx® test and serum creatinine, namely the CreLiMAx risk score. The benefits of the new CreLiMAx risk score were its high diagnostic accuracy and its potential to refine the assessment of end-stage liver disease (ESLD), beyond information provided by the MELD score.

In this validation study we aimed to reproduce the CreLIMAx diagnostic accuracy in patients with end-stage liver disease in liver related death within 12 months of follow-up.

## Methods

### Study design

The present analysis is based on data derived from a prospective observational study. Patients with ESLD were recruited via gastroenterological and surgical outpatient and inpatient departments between May 2012 and July 2014. The study was approved by the Charité Institutional Ethics Committee and registered at the German Clinical Trials Register prior study start (DRKS-ID: DRKS00005308).

### Study concept

At the time of study enrolment clinical examinations and routine laboratory tests were performed in all patients in order to calculate predictive models. In addition, patients underwent two additional tests on the day of enrolment (range 0–7 days) to assess the degree of liver fibrosis: an enzymatic liver function test [maximum liver function capacity (LiMAx®) test], and transient elastography (Fibro Scan®).

Study participants were followed up for 1 year from enrolment or until a study endpoint (death or liver transplantation) was reached. Causes of death were assigned by study personal to three categories: liver-related (*n* = 13), not liver-related (*n* = 2) unclear (*n* = 2).

### Study population

Survival data of 113 patients with ESLD were assessed. Inclusion criteria were a biopsy proven cirrhosis or clinical signs of cirrhosis [presence of at least two of the following complications: history of encephalopathy, liver associated ascites, endoscopically proven oesophageal varices, cirrhosis suggested in imaging (irregular shape of the liver, spleenomegaly)] or biochemical signs of cirrhosis [platelet count < 120/nL in the absence of a hematologic disorder, INR greater than 1.5 and albumin levels lower than 30 g/L]. Exclusion criteria for the analysis comprised previous liver surgeries including liver transplantation (LTx), the necessity for liver support therapy, parenteral nutrition, recent and past drug consumption, suspected or diagnosed hepatocellular carcinoma, acute on chronic liver failure and prior insertion of a transjugular intrahepatic portosystemic shunt. Written informed consent was obtained from all enrolled patients.

Finally, in order to address the accuracy of diagnostic tests in patients with Child-Pugh B, we performed a subgroup analysis in patients with Child-Pugh class B cirrhosis at the time of study enrolment (*n* = 41).

## Methods

MELD [2] and MELD-Na [3] were calculated according to previously described formulas. The recently proposed CreLiMAx risk score was calculated according to the authors’ previously published work [15]. The Child-Pugh score (CPS) was computed according to a published formula [1]. Encephalopathy was classified in 4 grades according to the West-Haven Criteria [16], presence and severity of ascites was being graded according to published guidelines [17].

The individual enzymatic liver function of the patient was assessed with the LiMAx® test. The test methodology is grounded on a 2 mg/kg bodyweight-adjusted intravenous ^13^C-labeled methacetin administration and subsequent liver-specific metabolisation of the substrate via the CYP 1A2 isoenzyme complex. The emerging ^13^CO_2_/^12^CO_2_ ratios are continuously measured by means of online breath-sampling using a special isotope-selective infrared spectroscope (FLIP, Humedics GmbH, Berlin, Germany). Values > 315 μg*/kg/h* are considered normal.

### Statistical analysis

Data were analyzed using the IBM SPSS Statistics 24 software (IBM SPSS Statistics for Windows, Version 24.0. Armonk, NY: IBM Corp.) and SigmaPlot Version 13 (Erkrath, Germany: Systat Software GmbH). In order to analyze the pattern of missing values we applied Little’s Missing Completely at Random (MCAR) test. In total missing values were found for 4 variables [Ascites grade 1 (0.9%) missing values; Bilirubin 5 (4.4%) missing values; INR 4 (3.5%) missing values; platelet count 4 (3.5%) missing values]. To account for missing values, an expectation maximization technique was used to impute missing observations. Continuous variables are shown as median and interquartile range (IQR; 25th–75th percentile). Categorical variables are shown as frequencies and percentage. The chi-squared test was applied for categorical variables and the Mann–Whitney *U*-test to compare quantitative variables. Correlations were calculated using the Spearman correlation coefficient. In order to assess the results of selected parameters with the best sensitivity and specificity to discriminate between patients who survived and those who either underwent LTx or died due to liver failure during the 1-year follow-up, we used the receiver operating characteristic (ROC) curve analyses and calculated the area under the ROC curve (AUROC). Optimal cut-offs were determined by the highest Youden index (corresponding to the sum of sensitivity and specificity minus 1). Patients with unclear and non-liver-related death were censored for the 12-month analysis (4 patients died after the 6-month follow-up). Differences between paired ROC curve area comparisons was performed using the DeLong test [18] In order to assess the multiple testing error we performed ANOVA with Bonferroni correction in our subgroup analysis. Survival curves were estimated using the Kaplan-Meier method for with patients censored at the time of transplantation or non liver-related death. Positive diagnostic likelihood ratios (LR) were used to evaluate the survival probability of patients at risk; values greater than 1 increasing the probability of disease [19]. A *p*-value of less than 0.05 for two sided tests was considered statistically significant.

## Results

### Study population

**Fig. 1:**
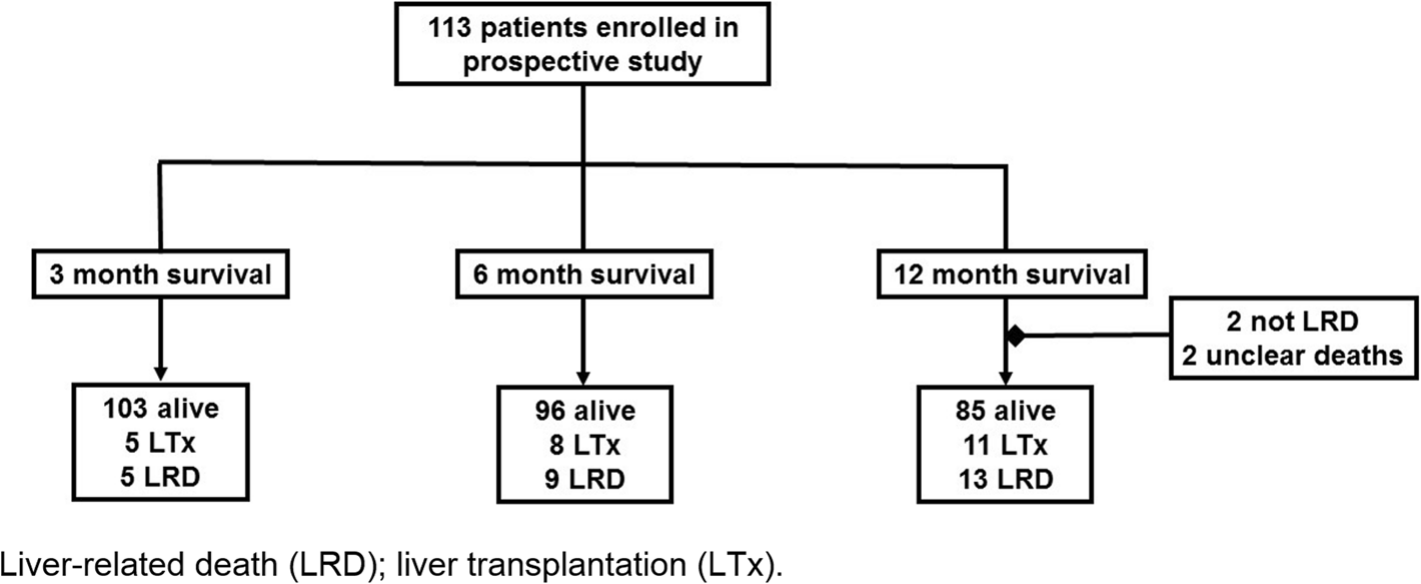
Flow Chart of patient inclusion for this study with respect to survival analysis

Clinical and epidemiological characteristics of the overall cohort are depicted in Table 1. Alcoholic liver disease was the main cause of liver disease (*n* = 51; 45%). Within 12 months of follow-up 11 patients (9.7%) underwent LTx and 17 (15.1%) died. Among these 13 deaths were liver-related, while two were not liver-related (spontaneous intracranial bleeding and subarachnoidal bleeding after downfall). In two patients the cause of death remained unclear (Fig. 1). The time between study enrolment and death was 142 (45–235) days and 91 (57–237) days between study enrolment and LTx.

**Table 1:** Epidemiological, clinical and biochemical characteristics of 113 patients with end-stage liver disease within a follow-up period of 12 months

Variables	Patient Cohort
Age [yrs]	58 (51–63)
Gender n (%)	
female	52 (46.0)
male	61 (54.0)
BMI [kg/m^2^]	28.0 (23.9–31.1)
Aetiology n (%)	
alcoholic	51 (45.1)
cholestatic	15 (13.3)
NAFLD	8 (7.1)
viral	13 (11.5)
others	26 (23.0)
Serum albumin [g/L]	36.2 (32.4–40.9)
Serum bilirubin [g/dL]	1.2 (0.7–2.6)
INR	1.30 (1.14–1.51)
Platelet count [/nL]	111 (75–152)
Serum creatinine [mg/dL]	0.88 (0.74–1.24)
Serum sodium [mmol/L]	139 (136–141)
Ascites grade n (%)	
none/mild	82 (72.6)
moderate	11 (9.7)
severe	20 (17.7)
HE grade n (%)	
grade 0	71 (62.8)
grade I	39 (34.5)
grade II	2 (1.8)
grade III	1 (0.9)
Oesophageal varices n (%)	
yes	77 (68.1)
no	35 (31.0)
unknown	1 (0.9)
Previous GI haemorrhage n (%)	28 (25.7)
Child-Pugh classes n (%)	
A	53 (46.9)
B	41 (36.3)
C	19 (16.8)
Fibroscan stiffness [kPa]	38.5 (20.5–59.3)
MELD	12 (9–16)
MELD-Na	13 (9–19)
CreLiMAx score	0.94 (0.91–0.96)
LiMAx® [μg/kg/h]	165 (104–273)
Underwent LTx n (%)	11 (9.7)
Liver-related death n (%)	13 (11.5)

*BMI* Body Mass Index; *CreLiMAx* Creatinin-LiMAx risk score; *HE* hepatic encephalopathy; *GI* gastrointestinal; *INR* international normalized ratio; *M* months; *MELD* model of end-stage liver disease; *LiMAx®* maximum liver function capacity; *LTx* liver transplantation; *NAFLD* non-alcoholic fatty liver disease

### Diagnostic accuracy

The LiMAx® test showed a significant negative correlation with the MELD score (r_s_ = − 0.65; *p* < 0.001) and CPS (r_s_ = − 0.62; *p* < 0.001). Also, the newly proposed prognostic CreLiMAx risk score correlated significantly with MELD (r_s_ = − 0.72; *p* < 0.001) and CPS (r_s_ = − 0.55; *p* < 0.001, respectively). At the time of study enrolment, mathematical models as well as the LiMAx® test were significantly different between patients who survived and those who died due to liver-failure related complications within 12 months (data not shown).

We determined the best discriminative cut-off values to predict the probability of liver-related death in our cohort. This confirmatory analysis showed similar results for the estimated 3-month survival when compared to data deriving from a different cohort of ESLD-patients recently published by the authors.

The current results show that the CreLiMAx risk score addresses short-term and medium-term mortality risk with comparable diagnostic accuracy. Positive diagnostic likelihood ratios (LR) (values greater than 1 increase the probability of disease) were best for the CreLiMAx risk score, also with the best diagnostic accuracy for the evaluation of the survival probability of patients at risk. Table 2 summarizes values of diagnostic accuracy.

**Table 2:** Cut-off values and accuracy of diagnostic models for liver-related survival for 3, 6 and 12 months for the entire cohort (*n* = 113)

Variable	Cut-off	Sensitivity	Specificity	PPV	NPV	Positive LR	Negative LR	Accuracy
**3 M**								
MELD	15	0.80	0.75	0.13	0.99	3.17	0.27	0.75
MELD-Na	15	0.80	0.68	0.11	0.99	2.50	0.29	0.69
CreLiMAx	0.9	0.60	0.82	0.14	0.98	3.25	0.49	0.81
**6 M**								
MELD	15	0.88	0.78	0.25	0.99	4.00	0.16	0.79
MELD-Na	15	0.89	0.72	0.23	0.99	3.16	0.15	0.73
CreLiMAx	0.9	0.56	0.84	0.25	0.95	3.56	0.53	0.82
**12 M**								
MELD	15	0.69	0.80	0.35	0.94	3.46	0.38	0.79
MELD-Na	15	0.85	0.75	0.34	0.97	3.42	0.20	0.77
CreLiMAx	0.9	0.54	0.87	0.39	0.93	4.16	0.53	0.83

Creatinin-LiMAx risk score (CreLiMAx); months (M); model of end-stage liver disease (MELD); maximum liver function capacity (LiMAx); Likelihood ratio (LR); negative predictive value (NPV); positive predictive value (PPV)

### Survival analyses

AUROCS for 3-month liver related risk of death were for MELD [0.86 (95% CI 0.72–1.00)] *p* = 0.007; MELD-Na [0.89 (95% CI 0.77–1.00)] *p* = 0.003; CPS [0.86 (95% CI 0.77–0.94) *p* = 0.007] and CreLiMAx score [0.81 (95% CI 0.73–0.97) *p* = 0.08].

**Fig. 2:**
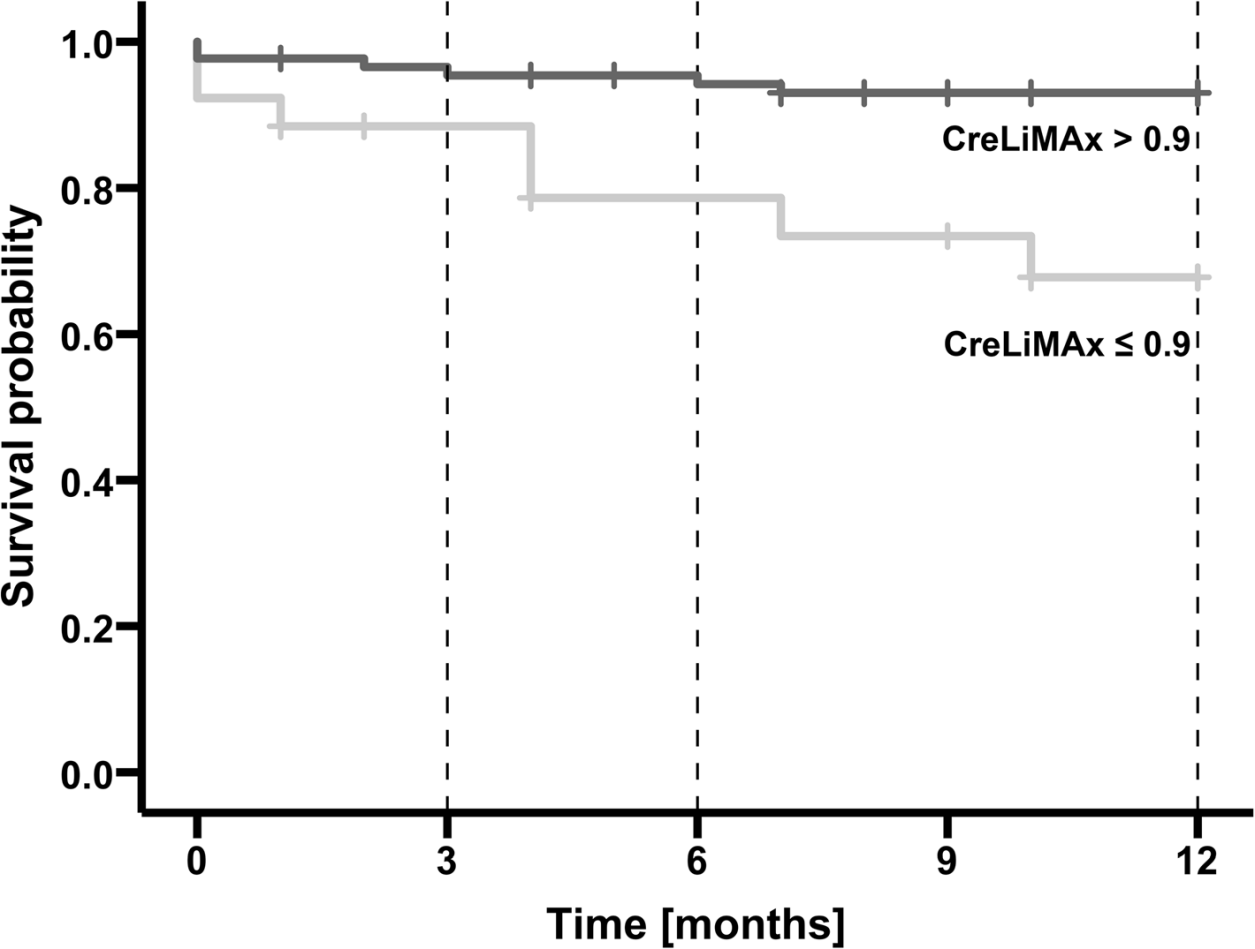
Kaplan-Meier estimates according to CreLiMAx risk score cut-off values. (patients were censored at time of transplantation or not liver-related death). The Breslow-Wilcoxon test was used to determine differences between survival curves: *p* = 0.223 (CreLiMAx risk score cut-off 0.9) for 3 months, *p* = 0.003 (CreLiMAx risk score cut-off 0.9) for 6 months and *p* = 0.002 (CreLiMAx risk score cut-off 0.9) for 12 months, respectively

Data on medium-term survival (6 and 12 months) showed similar patterns: MELD [AUROC 6 months 0.89 (95% CI 0.80–0.98) *p* < 0.001; AUROC 12 months 0.89 (95% CI 0.81–0.96) *p* < 0.001], MELD-Na [AUROC 6 months 0.93 (95% CI 0.85–1.00) *p* < 0.001 and AUROC 12 months 0.89 (95% CI 0.80–0.98) *p* < 0.001] and CPS [AUROC 6 months 0.91 (95% CI 0.85–0.97) *p* < 0.01 and AUROC 12 months 0.88 (95% CI 0.80–0.96) *p* < 0.001] showed higher concordance indexes when compared with the CreLiMAx risk score [AUROC 6 months 0.80 (95% CI 0.67–0.96) *p* < 0.01 and AUROC 12 months 0.79 (95% CI 0.64–0.94) *p* = 0.001]. Paired ROC curve area comparisons did not meet statistical significance for 3, 6 and 12 months, respectively (data not shown). Figure 2 shows survival stratified according to respective CreLiMAx risk score cut-off values.

### Subgroup analysis in patients with child-Pugh class B cirrhosis

Because of the known clinical heterogeneity of patients with Child-Pugh Class B cirrhosis we examined the prognostic ability of MELD, MELD-Na and the CreLiMAx risk score in this subgroup separately. Table 3 summarizes the clinical baseline data of these 42 patients.

**Table 3:** Epidemiological, clinical and biochemical characteristics of 41 patients with Child-Pugh Class B cirrhosis

Variables	Patient Cohort
Age [yrs]	55 (31–75)
Gender n (%)	
female	41.5% (17.0)
male	58.5% (24.0)
BMI [kg/m^2^]	27.2 (17.5–37.88)
Aetiology n (%)	
alcoholic	16 (39%)
cholestatic	6 (14.6%)
NAFLD	4 (9.8%)
viral	4 (9.8%)
others	11 (26.8%)
Serum albumin [g/L]	3.39 (2.51–4.98)
Serum bilirubin [g/dL]	3,3 (0.15–20.62)
INR	1.34 (0.91–1.79)
Platelet count [/nL]	125 (40–377)
Serum creatinine [mg/dL]	1.34 (0.39–8.90)
Serum sodium [mmol/L]	137.4 (127–145)
Ascites grade n (%)	
none/mild	24 (58.5%)
moderate	7 (17.1%)
severe	10 (24.4%)
HE grade n (%)	
grade 0	22 (53.7)
grade I	16 (39)
grade II	1 (2.4)
grade III + IV	2 (4.8)
Oesophageal varices n (%)	
yes	32 (78%)
no	9 (22%)
unknown	0
Previous GI haemorrhage n (%)	13 (31.7%)
Child-Pugh classes n (%)	
A	0 (0)
B	41 (100%)
C	0 (0)
Fibroscan stiffness [kPa]	38.5 (20.5–59.3)
MELD	14.6 (8–28)
MELD-Na	16.2 (7–28)
CreLiMAx	0.93 (0.89–0.95)
LiMAx [μg/kg/h]	184 (44–725)
Underwent LTx n (%)	3 (7.3%)
Liver-related death n (%)	7 (17.1%)

*BMI* Body Mass Index; *CreLiMAx* Creatinin-LiMAx risk score; *HE* hepatic encephalopathy; *GI* gastrointestinal; *INR* international normalized ratio *M* months; *MELD* model of end-stage liver disease; *LiMAx* maximum liver function capacity; *LTx* liver transplantation; *NAFLD* non-alcoholic fatty liver disease

**Fig. 3:**
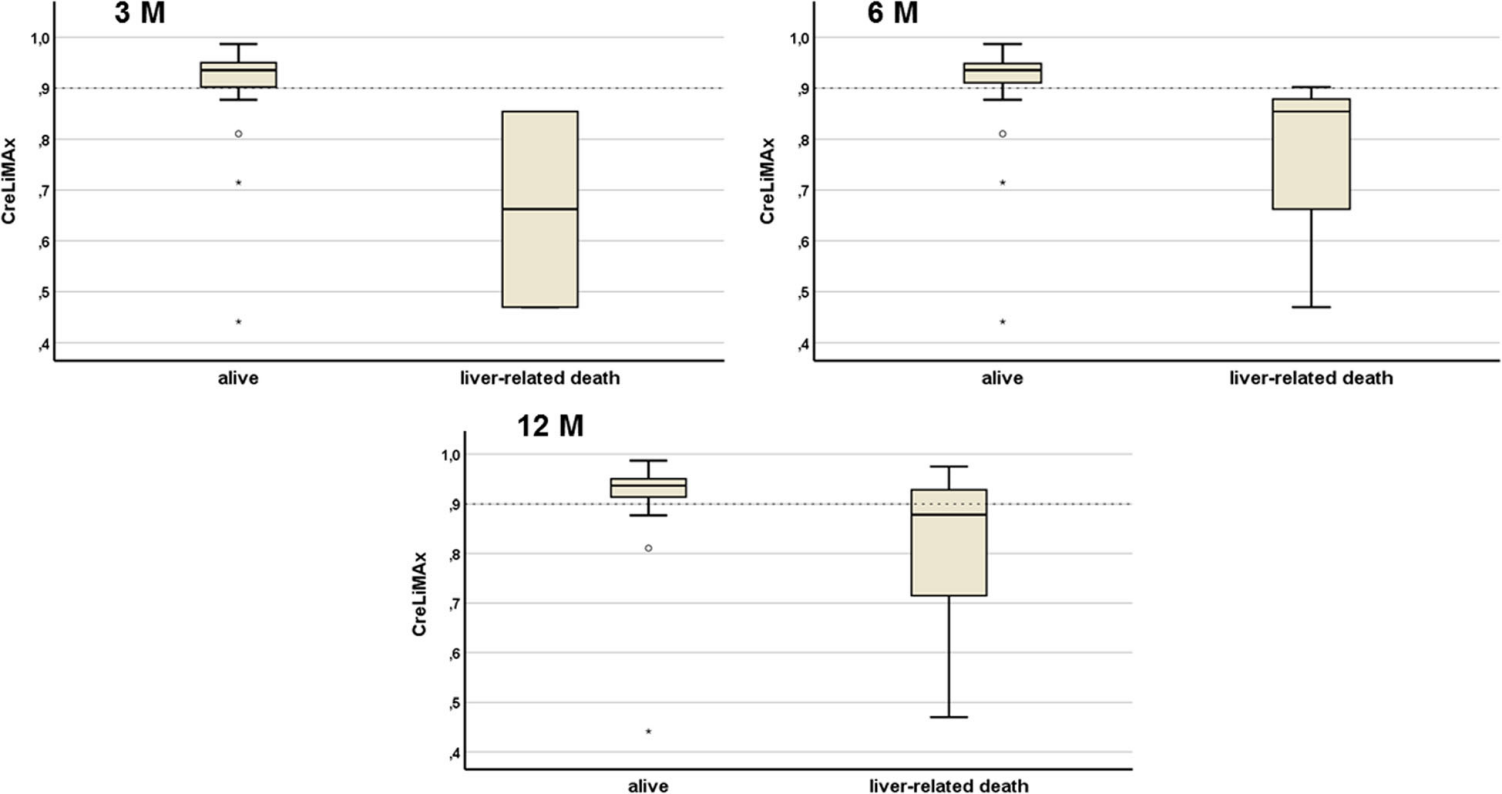
Outcome of patients according to the Cre-LiMAx risk score values measured on evaluation day. Medians are indicated by bold lines, the range from lower to upper quartile by boxes, 1.5 interquartile range by whiskers and outliers by circles. The cut-off (≤0.9points) is indicated by a bold dotted horizontal line. Differences between survivors and non-survivors were 0.91 (±0.1) and 0.66 (±0.27) points (*p* = 0.007) for 3-month survival; 0.91 (±0.1) and 0.74 (±0.24) points (*p* = 0.037) for 6-month survival and 0.91 (±0.1) and 0.81 (±0.19) points (*p* = 0.117) for 12-month survival (patients with unclear and non-liver-related death [*n* = 4] were censored), respectively.

The CreLiMAx risk score remained the only parameter that significantly differed in non-survivors and survivors within this subgroup. Figure 3 shows Boxplot diagrams for 3, 6 and 12 months, respectively.

In this subgroup analysis measures of diagnostic accuracy were strong for the CreLiMAx score. Positive and negative LR were best for the CreLiMAx score, again with best diagnostic accuracy for the evaluation of the survival probability of patients at risk. Table 4 summarizes values of diagnostic accuracy.

**Table 4:** Cut-off values and accuracy of diagnostic models for liver-related survival for patients with Child-Pugh Class B cirrhosis (*n* = 41)

Variable	Cut-off	Sensitivity	Specificity	PPV	NPV	Positive LR	Negative LR	Accuracy
**3 M**								
MELD	15	1.0	0.66	0.13	1.0	2.92	0.00	0.68
MELD-Na	15	0.50	0.50	0.50	0.95	1.0	1.0	0.50
CreLiMAx	0.9	1.0	0.76	0.18	1.0	4.22	0.00	0.78
**6 M**								
MELD	15	0.67	0.66	0.14	0.96	1.94	0.51	0.66
MELD-Na	15	0.67	0.51	0.11	0.95	1.37	0.65	0.53
CreLiMAx	0.9	0.67	0.77	0.20	0.96	2.92	0.43	0.76
**12 M**								
MELD	15	0.50	0.67	0.23	0.87	1.50	0.75	0.64
MELD-Na	15	0.67	0.53	0.22	0.89	1.43	0.63	0.56
CreLiMAx	0.9	0.50	0.80	0.33	0.89	2.50	0.62	0.75

*CreLiMAx* Creatinin-LiMAx risk score; *M* months; *MELD* Model of end-stage liver disease; *LiMAx* maximum liver function capacity; *LR* Likelihood ratio; *NPV* negative predictive value; *PPV* positive predictive value

AUROCS for 3-, 6- and 12-month liver-related risk of death for the CreLiMAx risk score were [0.95 (95% CI 0.87–1.00) *p* = 0.04; 0.89 (95% CI 0.76–1.00) *p* = 0.03; 0.74 (95% CI 0.49–0.99) *p* = 0.07], MELD [0.86 (95% CI 0.64–1.00) *p* = 0.09; 0.78 (95% CI 0.57–0.99) *p* = 0.11; 0.75 (95% CI 0.58–0.92) *p* = 0.06] and MELD-Na [0.72 (95% CI 0.33–1.00) *p* = 0.29; 0.74 (95% CI 0.47–1.00) *p* = 0.17; 0.67 (95% CI 0.42–0.92) *p* = 0.13]. Paired ROC curve area comparisons did not meet statistical significance for 3 months, 6 months and 12 months, respectively (data not shown).

## Discussion

Results emerging from the present study confirm the CreLiMAx risk score as a valid estimate of 3-month survival for patients with ESLD. Moreover, the new score was confirmed as an eligible predictor of 6- and 12-month survival, with good discriminative ability to identify transplant candidates at risk of dying within this period.

Initially, the LiMAx® test has been reported as a reliable method to evaluate actual liver function and to predict postoperative outcome in the perioperative work-up of patients with liver pathologies [11, 20]. Furthermore, the authors were able to demonstrate its prognostic accuracy in patients with acute liver failure [12]. In addition, we were able to demonstrate that the LiMAx® test is an appropriate marker for 6-month survival prognosis in liver transplant candidates [21]. Supported by these finding we recently proposed the CreLiMAx risk score for the evaluation of short-term mortality risk in patients with chronic liver disease [15].

In our current analysis we were able to confirm the ability of the new score to estimate short term survival in an independent prospective cohort study. Compared with our initial publication, this confirmatory study comprised a similar set of patients and comparable numbers of liver-related deaths and liver transplantations performed.

Additionally, in this study we were able to demonstrate the potential of the CreLiMAx risk score in predicting medium-term mortality. Although, measures of diagnostic accuracy for all clinically relevant models were comparable, positive likelihood ratio and diagnostic accuracy were slightly higher for the CreLiMAx risk score. Likelihood ratio is commonly seen as being less likely to change with the prevalence of the disorder and thus has an advantage over sensitivity and specificity [22]. Hence, the positive likelihood ration of 3.25 means a CreLiMAx risk score < 0.9 is 3 times as likely to be seen in a patient who survives, than in a patient who dies within 3 months. In contrast, MELD focuses on identifying the sickest patients with the highest risk of death. Therefore, both tools might be used in a synergetic manner to estimate outcome in such patients.

A recent study aimed to demonstrate the benefit of the incorporation of liver function tests into existing survival scores to improve diagnostic accuracy [23]. However, linkage to preexisting scores was criticized as being one of the major drawbacks. Several other studies have already shown that liver function tests per se are capable of estimating survival. To our knowledge, no study has yet been able to convincingly demonstrate prognostic superiority to established scores. In 2013, Giannini et al. showed the ability of a breath test to distinguish between different degrees of liver function impairment and clinical and histological findings in cirrhotic patients [24]. A large prospective study reported a similar - but not superior - diagnostic power of liver tests when compared with established tests to estimate disease related survival [25]. Stravitz et al. recently suggested that a liver specific breath test could better classify risk of cirrhotic complications and mortality than certain MELD thresholds [10]. Although the study cohort mainly contained patients with mild liver impairment, CPS was not considered as a method of reference. Over decades CPS has been widely used as the reference for assessing outcome in cirrhotic patients [1, 26]. However, within early stages of compensated liver disease, scoring systems - such as CPS and MELD - provide only limited prognostic value for short-term survival [27]. Furthermore, a recent systematic review examined the differences in survival predictions of CPS versus MELD in regard to different clinical settings [5]. Especially within the strata of early CP classes, algorithms based on knowledge of true liver function may provide additional information for a refined patient treatment. The LiMAx® test has already been proven to reflect true residual organ function otherwise not detected by routinely used methods [14]. This appears to be of great importance for the estimation of disease severity and the outcome prediction of patients with chronic liver disease. In particular, Child-Pugh Class B comprises patients with a high degree of heterogeneity with respect to residual liver function. In this stratum, patients with lower CPS but severely decreased liver specific test results might have a poor prognosis comparable to patients with higher CPS, despite the absence of clinical complications. In our study, the CreLiMAx risk score showed a good predictive performance in the Child-Pugh Class B. It was the only test that significantly predicted the discrimination between patient survival and death within the strata of the CPS. In particular, the CreLiMAx risk score was characterized by an excellent negative predictive value and a very good positive as well as negative likehood ratio.

This study is of course not the first evaluating a new score to stratify patients with cirrhosis according to their prognosis. However, to our best knowledge this is the first confirmatory study that evaluates the performance of an enzymatic-based liver function model for disease severity.

Certainly, our study has limitations. Although the number of primary endpoints (number of transplantations / number of patient deaths) were comparable in both the initial and confirmatory studies, cohorts differed in some clinical characteristics. The small difference seen in etiology, baseline demography and disease severity reflects the variety in epidemiology of patients with chronic liver disease. Secondly, diagnostic accuracy of disease severity scores differed only marginally. Our analysis strongly focuses on Child-Pugh Class B patients. We must admit that by performing this subgroup analysis the MELD might underperform in estimating patient survival when compared with the CreLiMAx risk score. Since patients’ values for serum bilirubin and INR are parameters of the MELD and CPS equation, mathematical bias may be present.

In any case, the CreLiMAx risk score showed a good ability to identify patients with low risk of death. Therefore, the CreLiMAx risk score seems to be a valid tool for providing additional information for a holistic clinical decision-finding in patients facing an allocation system based on a sickest-first policy.

## Conclusion

In conclusion, the study confirmed the CreLiMAx risk score to be a competitive and valid tool for estimating not only short-, but also medium-term survival in patients with end-stage liver disease. In particular in patients with Child Pugh Class B cirrhosis the new score best identified patients who were not at risk of death and might represent a complementary tool to the MELD for the assessment of the outcome of patients with chronic liver disease.

## Data Availability

The datasets used and/or analysed during the current study are available from the corresponding author on reasonable request.

## Abbreviations

AUROC: Area under the receiver operating characteristics
BMI: Body Mass Index
CI: Confidence interval
CPS: Child-Pugh score
CreLiMAx: Creatinin-LiMAx risk score
ESLD: End-stage liver disease
HE: Hepatic encephalopathy
INR: International normalized ratio
IQR: Interquartile range
LiMAx: Maximum liver function capacity
LR: Likelihood ratio
LTx: Liver transplantation
MELD: Model for end-stage liver disease
MELD-Na: Sodium MELD
NAFLD: Non-alcoholic fatty liver disease
NPV: Negative predictive value
PPV: Positive predictive value
ROC: Receiver-operating characteristic
SD: Standard deviation
